# Medical News and Items

**Published:** 1878-04

**Authors:** 


### Medical News and Items.

Rush Medical College.—The Thirty-fifth Annual Commencement exercises of this college were held in the Jefferson Park Presbyterian Church, February 26th, 1878. After prayer by the Rev. Francis L. Patton, D.D., President J. Adams Allen conferred the degrees upon 129 gentlemen who passed their examination to the satisfaction of the Faculty, out of the 141 who originally entered the graduating class. One honorary and four *ad eundem* degrees were also conferred—133 in all. The charge to the graduates by the President was followed by the response on behalf of the class by Dr. A. C. Cotton, after which Prof. Chas, T. Parkes delivered the Valedictory address. We regret our inability to overcome Dr. Parkes' modesty to secure his address for publication.

The following are the names of the graduating class:

Arnold, Edward Dudley ; Alford, James Simpson ; Anderson, Jeremiah Allen ; Abrams, James Henry ; Brattain, Benjamin Franklin ; Baldwin, Aristides Edwin ; Bowman, Andrew Washington ; Burnham, Alonzo Festus; Burlingame, John Henderson ; Boyd, Robert Dempsey; Browne, Alfred Marshman; Brown,

Commodore Perry; Bergen, George Matthews, A. B.; Burton, Daniel Francis, B.S.; Barry, John Samuel; Butz, John Edmund P.; Bennett, Edwin George; Brainerd, Henry Green; Burhans, Orvis Mann; Boardman, Edwin Orlando; Boganau, Sau; Bates, Frederick Herbert; Bellus, George Wesley; Bullard, Francis Bascom; Bond, Arthur Grant; Currens, John Randolph; Carlton, Lewis William; Cotton, Alfred Cleveland; Culver, Jacob; Craig, Augustus Lessure; Cram, Fred Warren; Christiancy, Victor Hugo; Camp, James Leeworthy, Jr.; Dawley, George; Darrow, Edward McLaren; Dinsdale, James, A.B.; Dewey, James J.; DePuy, Ozias; Demsey, Cyrus Felix; Daniels, William Nehemiah; Eldridge, Frank Paris; Ford, Lyman Washington; Forhan, Thomas Joseph; Furber, William Warren; Fieldhouse, James; Ferris, Charles Leonard, A.B.; Garrey, John Eugene; Godfrey, Byron Benjamin; Glennan, Michael Augustine; Goldspohn, Albert, B.S.; Hicks, Levi Nevada; Hobart, Jefferson Roger; Hall, William Edward; Hall, Joseph H.; Hurd, Herbert Halsey, A.B.; Hathaway, Lawrence Bryant; Hardman, Charles; Hinde, Alfred; Hayman, Lucius Henry; Hewitt, Henry Miller; Irwin, Judson Deforrest; Isherwood, Hortensus Lowry; Johnson, Austin H.; Kelly, Elijah Stephens; King, William Henry Kane; Kemper, Phillip Amiss; Logan, John Augustus; Long, Charles Melville; Murphy, John Redfield; Murphy, William Thomas; Morse, Ashbel Henry; McHugh, Uriah Clay; Miller, Samuel Ross; Mailer, Andrew Caldwell; McCoy, Hiram Foster; Miller, Samuel Borland; Metzradt, Hans Von; McClelland, Robert Alexander; Mills, Aaron; Major, Elverton E.; Nolan, Emanuel Cross; O'Conner, John Chrysler; Patterson, Fred William; Porter, Epaphroditus Jehoshaphat; Pratt, Howard Lewis; Pritchett, Gilbert Lafayette; Phillips, James Henry; Park, Henry Hull; Pettijohn, Abra Claudius; Porter, Dennis Wilson; Porter, Walter Howard; Quinn, Edward; Rathbun, Isaac Hale; Rathbun, Addison Milton; Rakenius, Hermann; Reed, Charles Corneau; Reid, Duncan, Ph. B.; Robinson, Andrew Jackson; Rogers, Talcott Austin; Reynolds, Emery Eugene; Ryon, George; Rounseville, Albert Parker; Sansom, Joseph Emmet; Sage, John B.; Sether, Christian; Shaw, James Emmet; Stretch, Ethan McAferty; Smolt, Charles

Frederic, B. S.; Stuart, George; Sexton, Albert Germain; Sherwin, Frank Oliver; Smith, William Lloyd; Salisbury, Jerome Henry, A. B.; Scott, James Edwin; Stiver, William Bike; Thayer, Carmi Casander, B.D.; Wilson, William Dean; Webb, Benjamin Oliver; Wheelwright, William Simmons; Wheeler, Edward Newby; Wear, Isaac Newton; Watson, Colin Christopher; Weens, Elwood; Woodbridge, Windsor Pelton; Wadhams, Frederick Eugene; Whitney, Eugene Wolcott; Wolfe, Albert Polk; Young, Vincent Phelps.

*Ad Eundem.*—Dr. John E. Owens, Dr. Norman Bridge, Dr. James Nevins Hyde, Dr. D. J. Loring.

*Honorary.*—Dr. John Burgess Walker.

The Woman's Hospital Medical College of Chicago.—The Eighth Annual Commencement Exercises of this institution took place at the Clark street M. E. Church on Thursday evening, February 28, 1878. After an overture by Maj. Nevans' orchestra the exercises were opened with prayer by the Rev. E. P. Goodwin, D. D. After which the Degree of Doctor in Medicine was conferred upon the following ladies by the President, Wm. H. Byford, A. M., M. D.: Ellenora Stallard, of Iowa; L. Anna Ballard, of Michigan; Helen B. Boddson, of Illinois; Clara Louisa Normington, of Illinois; Augusta Max Hyacinth, of Illinois; Nannie A. Stevens, of Illinois; Lida E. Green, of Illinois.

The Valedictory Address was then delivered by Prof. Wm. E. Quine, M. D. Subject, Courage.

A very fine audience was in attendance and the exercises passed off in an unusually pleasant manner, and were closed by the Benediction by the Rev. Dr. Goodwin.

On the following evening a very pleasant reception was given the class by Dr. and Mrs Byford, at their residence, 908 Indiana Av.

The Summer Course of Lectures will open on Tuesday, the 2d day of April, and continue till July 1st, '78.

Chicago Medical College.—The Nineteenth Annual Commencement exercises of this college were held in Plymouth Church (Michigan avenue, near 26th st.), on Tuesday, March 5, 1878. After prayer, the Dean of the College, Professor N. S. Davis, distributed certificates to undergraduates for hospital and dispensary attendance, awarded the college prizes, and conferred the degree of Doctor of Medicine upon the gentlemen whose names are subjoined. Professor H. A. Johnson delivered the annual address to the graduates, and Dr. M. S. Wylie responded for the class. Music was furnished by the organist, Mr. I. V. Flagler, and the Philadelphia Quartette Club. The exercises were concluded with the benediction.

Geo. B. Abbott, J. D. Andrew, R. H. Babcock, John S. Beers, B. F. Boyer, R. H. Broe, Wm. H. Byford, Wm. W. Cook, J. W. Dal, John Enlow, Wm. M. Farr, J. H. Fellows, Albert Green, A. J. Irwin, L. A. Irwin, D. L. Kenyon, Wm. R. Lawrence, M. S. Marcy, Jos. Matteson, Wm. H. McClain, J. W. McKibbin, J. E. McNeil, Geo. W. Moody, E. E. Moore, F. Mueller, Niels J. Nielsen, Frederic L. Nutt, W. F. Nye, Edward Pearce, N. Pierpoint, C. B. Richmond, C. J. Rivenburgh, Milton M. Rowley, J. L. Sawyers, John Schwendener, Henry C. Sibree, W. H. Smith, Wm. T. Speaker, Horace M. Starkey, L. A. Stearns, Ora F. Thomas, Frank E. Waxham, E. H. Webster, G. N. Wood, P. M. Woodworth, H. M. Workman, Mac S. Wylie.

*Honorary Degree.*—Emanuel Ridgway.

Discreditable Pictures.—An illustrated paper is published at Hot Springs, Arkansas, designed to advertise certain hotels and regular (?) physicians. These latter favor the public with their counterfeit presentments, done in wood, and glowing tributes to their own powers. They are getting a well-merited scoring from the Western medical journals, and it is to be hoped their discipline will not end with that. Nothing could well be conceived more unbecoming and discreditable.—*Phila. Med. and Surg. Reporter.*

